# Effectiveness of conventional surface water treatment processes in reducing natural radionuclides in Nile River drinking water

**DOI:** 10.1038/s41598-026-36428-y

**Published:** 2026-03-23

**Authors:** Khaled Ali, Zinab S. Matar, Shaban Harb, Khaled Salah El-Din, Clemens Walther, Mahmoud Kilany, Karem Moubark

**Affiliations:** 1 Physics Department, Faculty of Science, South Valley University, Qena, Egypt; 2 Physics Department, Collage of Science, Umm Al-Qura University, Makkah, Saudi Arabia; 3 Institute for Radioecology and Radiation protection, Leibniz University Hanover, 30419 Hanover, Germany; 4 Geology Department, Faculty of Science, South Valley University, Qena, Egypt

**Keywords:** Radon, Natural radionuclides, Water treatment, Nile River, Radiological safety, Environmental sciences, Hydrology

## Abstract

Understanding the stage-specific performance of conventional water treatment processes in removing natural radionuclides is crucial for optimizing public health protection, particularly in regions dependent on major river systems like the Nile. This study comprehensively evaluates the effectiveness of each treatment stage in conventional water treatment plants across Upper Egypt in reducing natural radionuclides, radon-222 (Rn-222), radium-226 (Ra-226), radium-228 (Ra-228), and potassium-40 (K-40), in Nile River-derived drinking water. We collected 40 water samples from 10 representative Nile-fed treatment plants in Upper Egypt, at 4 key stages: raw water intake, post-coagulation/sedimentation, post-filtration, and final treated water. Rn-222 concentrations were measured using the RAD7 detection system with RAD H_2_O accessory, while gamma-emitting radionuclides (Ra-226, Ra-228, and K-40) were analyzed via NaI(Tl) gamma spectrometry after achieving secular equilibrium between each parent and its short-lived progeny. The multi-stage conventional treatment process demonstrated differential effectiveness across radionuclides through distinct removal mechanisms. The treatment sequence achieved cumulative removal efficiencies (R_*eff*_) of 74.19% for Rn-222 through volatilization during various stages, 28.86% for Ra-226 through coagulation and particulate capture, 46.84% for Ra-228, and 20.17% for K-40, the lowest among the studied radionuclides, due to its predominantly dissolved ionic nature. Treatment stages contributed sequentially: coagulation removed 29.17% of Rn-222, 17.46% of Ra-226, 32.16% of Ra-228, and 11.85% of K-40; filtration further reduced Ra-226, Ra-228, and K-40, resulting in cumulative R_*eff*_ values of 26.06%, 42.04%, and 12.84%, respectively, by the end of this stage, while for Rn-222, filtration significantly enhanced its removal to a cumulative R_*eff*_ of 59.42%; final treatment (disinfection) achieved the aforementioned cumulative efficiencies for all radionuclides. The sequential multi-barrier approach resulted in calculated annual effective doses (D_*an*_) of 18.8 µSv/year (adults), 28.3 µSv/year (children), and 15.4 µSv/year (infants), all well below the international screening level of 100 µSv/year for a single source of radiation in drinking water, applicable to all age groups. These processes effectively mitigate radiological risks, with filtration being particularly crucial for volatile radionuclides and coagulation-filtration being essential for radionuclides that are associated with suspended particles, such as Ra-228. These findings provide critical insights for water treatment optimization and regulatory compliance in river-dependent communities.

**Supplementary Information:**

The online version contains supplementary material available at 10.1038/s41598-026-36428-y.

## Introduction

Water security remains a critical challenge for Egypt, where the Nile River serves as the lifeline for over 100 million people, providing the backbone for drinking water supply, agriculture, and industrial activities^1–3^. The reliability of this single-source dependency makes understanding and optimizing water treatment processes not plainly important, but essential for public health protection. Egyptian water treatment facilities employ conventional multi-barrier treatment systems, including coagulation, flocculation, sedimentation, filtration, and disinfection, primarily designed to eliminate microbial pathogens and suspended particles^1,4–6^. However, the effectiveness of these same processes in managing naturally occurring radioactive materials has received insufficient attention in national water quality frameworks. Natural radioactivity in surface waters primarily arises from 3 primordial radionuclides, uranium-238 (U-238), thorium-232 (Th-232), and potassium-40 (K-40), and the decay products of U-238 and Th-232. Among the naturally occurring radionuclides in drinking water, radium-226 (Ra-226) and radium-228 (Ra-228)—as soluble decay products of U-238 and Th-232, respectively—along with the gaseous radon-222 (Rn-222), typically represent the primary contributors to ingestion and inhalation doses under chronic exposure scenarios^7–15^. While uranium isotopes (e.g., U-238 and U-234) are known to contribute to natural radioactivity in aquatic systems, their direct measurement typically requires alpha spectrometry or ICP-MS, which were beyond the scope of this field-based assessment. Other naturally occurring radionuclides, such as lead-210 (Pb-210), and polonium-210 (Po-210), also contribute to radiological risk in drinking water, particularly through long-term ingestion pathways^16,17^. However, these nuclides require specialized analytical techniques, such as alpha spectrometry for Po-210 or low-background counting for Pb-210, that were not feasible within the scope of this large-scale, multi-stage field campaign. Our study focused on gamma-emitting radionuclides (Ra-226, Ra-228, K-40) and Rn-222, which are among the most commonly monitored species in conventional water treatment facilities due to their prevalence and public health relevance. Nevertheless, the presence of U-238 is implied by the observed levels of its decay progeny, Ra-226 and Rn-222, consistent with findings reported by the International Atomic Energy Agency (IAEA) in the context of natural radionuclide behavior in surface waters^18^. These contaminants enter aquatic systems through natural geological processes—leaching from bedrock, soil erosion, and mineral dissolution—making their presence inevitable rather than accidental^19,20^. Although concentrations are generally low, chronic exposure through daily consumption may pose health risks. Epidemiological evidence links chronic exposure to radionuclide-contaminated water to increased cancer risks: lung cancer is primarily associated with inhalation of Rn-222 released into indoor air during water use, while gastrointestinal cancers are linked to ingestion of Ra-226 and Ra-228^21–23^. While regional studies have assessed natural radioactivity in Egyptian groundwater^10,12,19,24^ and in Nasser Lake or Nile River^5,7,9^, a detailed, stage-resolved evaluation of radionuclide removal across the full sequence of conventional surface water treatment processes under real operational conditions in Upper Egypt is still lacking. Although general principles of radionuclide behavior in water treatment are documented^17,18^, there is a notable shortage of site-specific data tracking changes across coagulation, filtration, and disinfection stages in large river-fed systems. This knowledge gap creates uncertainty about whether existing treatment infrastructure adequately protects public health from radiological hazards. Understanding how each treatment stage, from raw water intake through final disinfection, contributes to radionuclide removal is crucial for both operational optimization and regulatory compliance. This study addresses this critical need by evaluating stage-specific removal efficiencies (R_*eff*_) of natural radionuclides in 10 representative water treatment plants across Upper Egypt. The findings offer evidence-based recommendations for water quality monitoring protocols and treatment process optimization, contributing to enhanced radiological safety standards for Nile-dependent populations.

## Experimental procedures

### Study area and water treatment plants

**Fig. 1:**
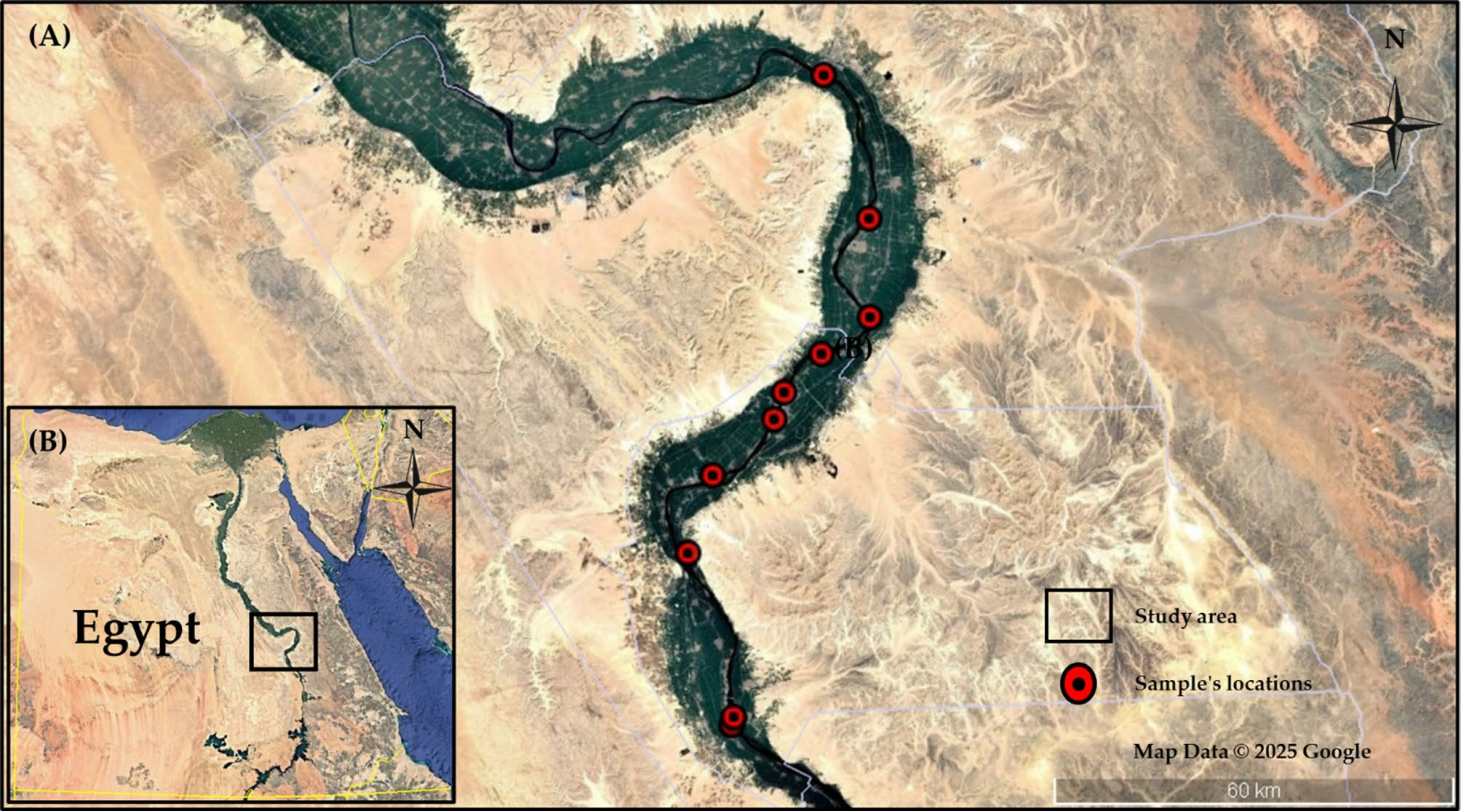
Geographic distribution of selected water treatment plants along the Nile River in Upper Egypt. The base map was created using Google Earth Pro Version 7.3^25^.

This investigation focused on 10 strategically selected Nile-fed water treatment facilities distributed across the central regions of Upper Egypt, specifically within Qena and Luxor governorates along the Nile Valley. These areas represent the heart of Upper Egyptian water infrastructure, where communities depend entirely on Nile River water for domestic, agricultural, and industrial needs. The selected plants serve as critical components of regional water supply networks, employing conventional multi-barrier treatment processes to prepare raw Nile water for public distribution. The typical treatment process in these Upper Egyptian facilities follows 4 distinct stages: (1) Raw water intake directly from the Nile River, where water is drawn through submerged pipes equipped with preliminary filters to prevent debris entry; (2) Coagulation stage, involving chemical addition (alum and primary chlorine) in large settling tanks to form flocs that capture suspended particles and associated radionuclides; (3) Filtration stage, utilizing specialized sand filters with fine mesh underdrains that allow only treated water to pass while retaining particulate matter and radionuclides; and (4) Final treated water stage, typically stored in ground-level contact tanks following chlorine disinfection. During this holding period, incidental aeration and degassing may further reduce volatile radionuclides such as Rn-222, though disinfection itself does not contribute to radionuclide removal. Site selection prioritized facilities that represent typical Egyptian treatment configurations while ensuring accessibility for comprehensive sampling at each treatment stage. To ensure scientific accuracy and reproducibility, all sampling locations were precisely documented using Global Positioning System (GPS) coordinates. The geographic distribution of these facilities is illustrated in Fig. 1, showing their strategic placement along the Nile corridor in central Upper Egypt. The Egypt map image was created using Google Earth Pro (Version 7.3)^25^. The study design emphasized comprehensive stage-by-stage analysis rather than inter-plant performance comparisons. This approach enabled detailed examination of how each treatment stage contributes to radionuclide R_*eff*_, providing practical insights for treatment optimization and regulatory compliance in Nile-dependent water systems.

### Samples collection and Preparation

Sampling was conducted over a 3-month period to account for potential variations in hydrological and operational conditions, while ensuring logistical feasibility across the 10 geographically distributed treatment plants. At each of the treatment facilities, a single but comprehensive sampling event was conducted during which water samples were systematically collected from 4 critical treatment stages: raw water intake, post-coagulation, post-filtration, and final treated water output. This approach ensured that all stages were measured under nearly identical operational conditions at each plant. Collection protocols followed strict quality assurance measures to preserve radionuclide integrity^26^. Samples were collected directly from main process lines using clean, air-tight high-density polyethylene bottles. Each container was filled completely with minimal agitation to prevent degassing and air pocket formation that could compromise Rn-222 concentrations. Immediate sealing and proper labeling with location, date, time, and treatment stage information ensured full sample traceability and enabled accurate temporal tracking across the sampling campaign^10^. For Rn-222 analysis, samples were transported in insulated containers to maintain low temperatures and minimize radioactive decay during transit. All samples were analyzed within 24 h of collection, with appropriate decay correction factors applied to account for storage and transportation periods. Gamma spectrometry samples required additional preparation for accurate radionuclide quantification. A separate set of water samples from each stage was transferred to pre-cleaned 1.4 L Marinelli beakers, previously rinsed sequentially with diluted hydrochloric acid followed by deionized water to minimize background contamination. These containers were hermetically sealed and stored for at least 30 days to establish secular equilibrium between Ra-226 and its progeny (e.g., Bi-214), and between Ra-228 and its short-lived progeny (Ac-228, Th-228, Pb-212) before gamma-ray analysis^9,12,13^. All procedures adhered to international standards for environmental radioactivity measurements, ensuring data quality suitable for radiological risk assessment^9,12,26^.

### Measurement instruments and techniques

Radionuclide analysis employed 2 primary analytical systems: the RAD7 detector and a NaI(Tl) gamma-ray spectrometer. The RAD7 system utilized pulse solid-state alpha detector technology for precise Rn-222 measurement through alpha particle detection during Rn-222 decay. Its established reliability in aqueous Rn-222 analysis made it suitable for comprehensive concentration assessments across all treatment stages. Gamma-emitting radionuclide quantification (Ra-226, Ra-228, and K-40) employed NaI(Tl) gamma-ray spectrometry. This technique identifies and quantifies isotopes by detecting characteristic energy peaks in gamma spectra. Samples were analyzed in sealed Marinelli beakers after 30-day storage periods to establish secular equilibrium between parent radionuclides and their short-lived progeny, ensuring accurate activity measurements. All instruments underwent proper calibration following manufacturer specifications and international standards before deployment. Measurements adhered to established protocols for environmental radioactivity analysis, maintaining data quality appropriate for radiological risk assessment. This dual-instrument approach enabled targeted evaluation of key gamma-emitting and gaseous radionuclides in the conventional treatment process.

#### RAD7 detector for Rn-222 activity measurements

**Fig. 2:**
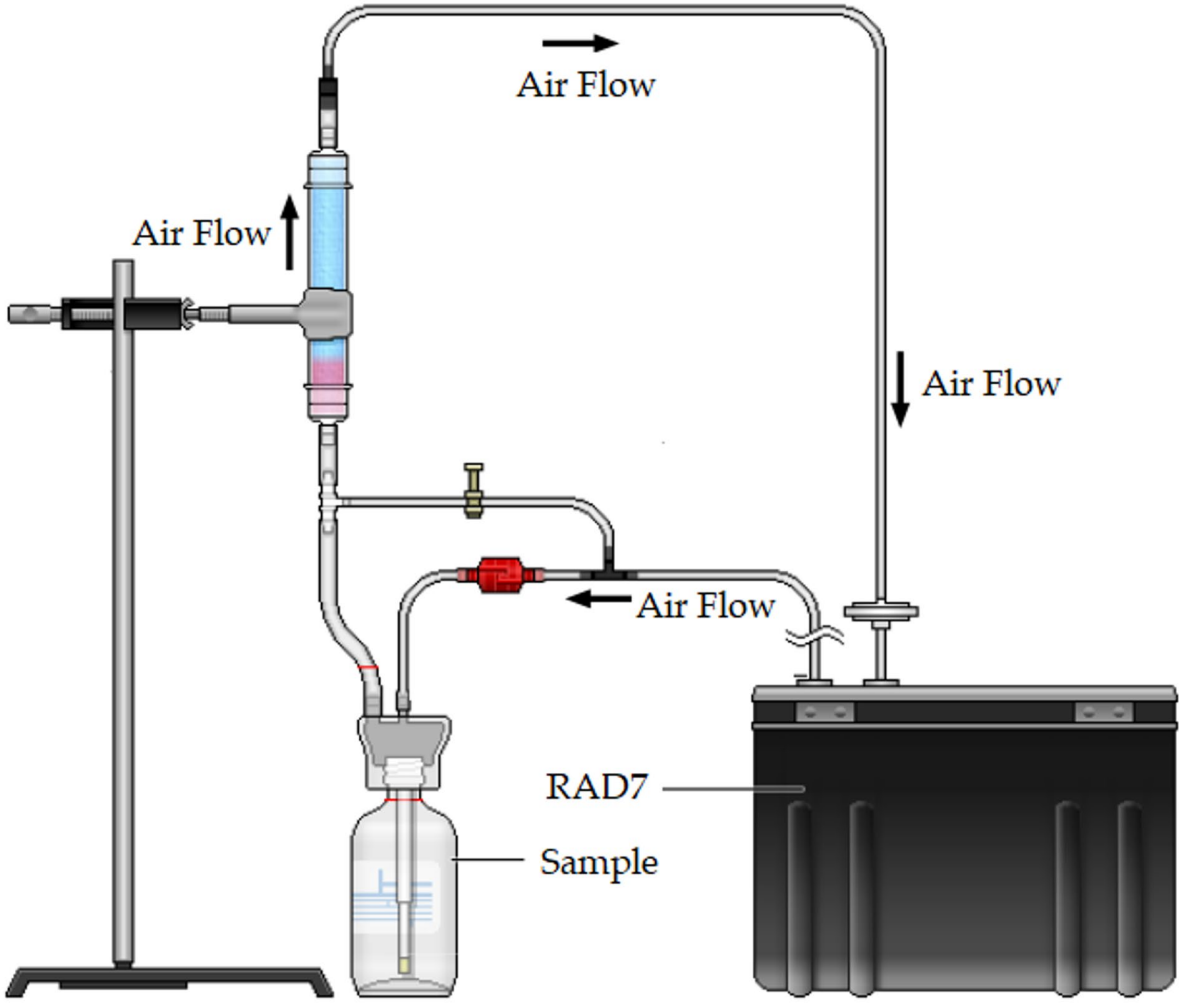
Schematic diagram of RAD7 H_2_O system configuration for Rn-222 measurement.

Rn-222 concentrations were determined using the RAD7 detector (Durridge Co., USA), a portable instrument designed for rapid measurement in water samples^27^. The system utilizes the RAD H_2_O accessory with closed-loop aeration methodology, minimizing Rn-222 loss during analysis while maintaining phase equilibrium. Sample analysis involved sealing 250 mL water samples in glass vials connected to the RAD7 unit. An integrated pump facilitated internal aeration for 5 min, transferring Rn-222 gas into the solid-state alpha detector where alpha particles from Rn-222 decay were detected by a silicon semiconductor detector. The Wat250 protocol governed all measurements, completing each analysis cycle in approximately 30 min: 5 min aeration (extracting over 94% of Rn-222), 5 min delay for progeny decay, followed by 4 consecutive 5-minute counting intervals. Final Rn-222 concentrations were calculated by multiplying the measured air-phase activity by a fixed conversion factor of 4, which accounts for the water-to-air volume ratio and the temperature-dependent partition coefficients. This factor is validated by the manufacturer for standard Wat250 analysis and ensures traceable quantification within the instrument’s operational range^27^. The RAD7 system has a Rn-222 concentration measurement range of 0.1–20,000 pCi/L (0.004–750 Bq/L), with an intrinsic background of 0.005 pCi/L (~ 0.185 mBq/L). According to the manufacturer, the EPA action level of 4 pCi/L (0.148 Bq/L) is detectable within 60 min with a 10% counting uncertainty. Given the 30-minute Wat250 protocol used in this study, the practical minimum detectable activity (MDA) for Rn-222 in water samples is estimated at 0.148 Bq/L, consistent with typical field deployment conditions. Real-time analysis capability minimizes storage requirements and decay corrections, making it particularly suitable for field applications. As shown in Fig. 2, the configuration creates a closed air loop between the RAD7 unit and sample vial, enabling efficient Rn-222 transfer and reliable measurement. The closed-loop aeration design minimizes Rn-222 loss and ensures representative measurement of the original sample concentration under field conditions^27–29^. The instrument provides a measurement accuracy of ± 5% under non-condensing humidity conditions (0–100% RH) when used with a desiccant. All reported Rn-222 concentrations are presented with no more than 3 significant figures, consistent with the instrument’s precision and the practical MDA.

#### Gamma-ray spectrometry using a NaI(Tl) gamma spectrometer for natural radionuclide analysis

Gamma-ray spectrometry for reliable quantification of Ra-226, Ra-228, and K-40 in water samples^9,10^. The technique identifies and quantifies target radionuclides by detecting their characteristic gamma-ray emissions, which serve as distinct spectral fingerprints^26^. The detection system consisted of a 3 × 3-inch NaI(Tl) scintillation detector (Model S-1212-I) coupled to a photomultiplier tube and a 1024-channel multichannel analyzer (Ortec Norland 5510). Operating at 805 V DC bias voltage, the system achieved an energy resolution of 7.5% at 662 keV and a peak efficiency consistent with the manufacturer’s specifications. A 50 cm thick lead shield was used to minimize background radiation, thereby enhancing measurement precision for low-level natural radioactivity. For measurement, water samples were transferred to 1.4 L Marinelli beakers that had been pre-cleaned by rinsing with dilute hydrochloric acid followed by deionized water. The samples were hermetically sealed and stored for at least 30 days to establish secular equilibrium between parent radionuclides and their short-lived progeny, a prerequisite for accurate Ra-226 and Ra-228 quantification, as per international guidelines^30^. Ra-228 activity was determined indirectly via its short-lived progeny Ac-228 (911.16 keV) and Pb-212 (238.6 keV), after 30 days of sealed storage to ensure secular equilibrium within the Ra-228 decay chain. It should be noted that Th-232 itself is not measurable in aqueous samples due to its extremely low solubility; thus, the reported activity reflects Ra-228. Each sample was counted for a minimum of 24 h to achieve adequate counting statistics. Energy calibration was performed using standard gamma sources, while detection efficiency was determined using multi-nuclide standards to enable precise quantification in Bq/L^30^. Radionuclides were identified by their characteristic gamma-ray energies: Ra-226 via the decay lines of Bi-214 (609.32, 1120.3, and 1765.5 keV); Ra-228 via Pb-212 (238.6 keV) and Ac-228 (911.16 keV); and K-40 directly by its 1460.82 keV emission. The MDA was calculated for each radionuclide and energy line based on Currie’s method^31^. The obtained MDA values were 0.067 Bq/L for Ra-226 (using the 609.3 keV line of Bi-214), 0.007 Bq/L (using the 1120.2 keV line), and 0.061 Bq/L (using the 1764.5 keV line); 0.010 Bq/L for Ra-228 (using the 238.6 keV line of Pb-212) and 0.009 Bq/L (using the 911.2 keV line of Ac-228); and 1.315 Bq/L for K-40. Although the mean K-40 concentration (3.71 Bq/L) was only approximately 2.8 times its MDA, the 24-hour counting duration and lead shielding ensured statistically robust quantification (net peak area > 3σ above background). Moreover, K-40 benefits from a prominent, isolated gamma peak at 1460.8 keV with high emission probability (10.7%), minimizing interference and enhancing detection reliability even at moderate activities. All reported activity concentrations exceeded their respective MDA values, confirming reliable detection. The activity concentration (A, in Bq/L) was calculated according to the following Eqs^9,10^.

$$\:A=\frac{C}{P\times\:V\times\:\epsilon\:}$$

where C is the net count rate (cps) corrected for background, P is the gamma emission probability, V is the sample volume (L), and *ε* is the system detection efficiency. Spectral analysis was performed using GENIE-2000 software for peak identification, background subtraction, and net count rate calculations. Given the moderate energy resolution of the NaI(Tl) detector, potential peak overlaps—particularly between the 583 keV line of Tl-208 (from the Th-232 series) and the 609 keV line of Bi-214 (from the Ra-226 series)—were evaluated using the built-in peak deconvolution algorithms in the GENIE-2000 software. All spectra were visually inspected, and overlapping peaks were resolved through Gaussian fitting routines based on known energy positions and relative intensities. The primary quantification lines (609.3 keV, 1120.3 keV, and 1765.5 keV for Ra-226; 338.4 keV and 911.2 keV for Ac-228; and 1460.8 keV for K-40) were selected for their minimal interference and were well separated from neighboring peaks under the employed measurement conditions. Measurement uncertainty was estimated by combining the statistical counting error, detector efficiency calibration uncertainty (± 5%), and sample volume measurement error (± 2%). The combined standard uncertainty ranged from 6% to 12%, depending on the radionuclide activity level. All values reported in the tables include the corresponding expanded uncertainty. This methodology enabled simultaneous multi-radionuclide detection with high accuracy, supporting a comprehensive radiological assessment of the treated Nile River water.

## Results and discussion

### Raw water intake baseline concentrations

The Nile River water entering treatment facilities in Egypt exhibited measurable levels of natural radionuclides, establishing baseline concentrations that reflect the geological characteristics of the river system. Comprehensive analysis of radionuclide concentrations across all treatment stages is presented in Table 1, which summarizes mean values, standard deviations, and R_*eff*_ for each radionuclide at every treatment stage. Detailed individual measurements for all samples are provided in Table S.1 in the supplementary materials. Spatial distribution and graphical representations illustrating radionuclide patterns are available in Figures S.1 in the supplementary materials. Mean concentrations in raw Nile water were 4.51 ± 0.23 Bq/L for Rn-222, 0.115 ± 0.01 Bq/L for Ra-226, 0.021 ± 0.002 Bq/L for Ra-228, and 3.71 ± 0.35 Bq/L for K-40 — all well below the World Health Organization (WHO) guidance levels for drinking water. The activity concentrations of natural radionuclides in raw Nile River water were found to be relatively low compared to other regional water sources across Egypt. To place these findings in context, Supplementary Table S.2 has been expanded to include not only local Egyptian data but also recent measurements from multiple countries within the Nile Basin, including Sudan^32^, Ethiopia^33,34^, Kenya^35^, Uganda^36^, and Tanzania^37^, alongside international studies from other major river systems^14,15^. This broader comparison confirms that the Upper Nile in Egypt exhibits among the lowest natural radioactivity levels reported across the basin, likely due to its sedimentary geology, dilution effects, and distance from uranium- or thorium-enriched crystalline terrains. Locally, our Ra-226 levels are significantly lower than those previously reported in Nasser Lake^9^, Qena groundwater^12^, and Safaga-Quseir^24^. Similarly, Ra-228 concentrations in raw Nile water are relatively low, consistent with the general trend of reduced dissolved radionuclide content in large, sediment-laden river systems compared to groundwater sources in the same region. For K-40, our measurements fall within the range observed in Qena groundwater^12^ but are higher than those reported for Nile water in Assiut^38^. These comparisons suggest that the Upper Nile region has relatively low background natural radioactivity, which contributes to the overall effectiveness of conventional treatment processes in producing radiologically safe drinking water. These observed baseline values are also consistent with patterns reported in other large river systems worldwide, where geological leaching and mineral dissolution govern the dissolved radionuclide content — though with notable variations depending on bedrock composition and hydrological dynamics^39^. The standard deviation for Rn-222 in raw water (± 0.23 Bq/L, ~ 5% of the mean) indicates good spatial and temporal consistency across sampling sites, despite its inherent volatility. Larger variability was observed in post-coagulation samples, likely due to differences in aeration during chemical mixing. R_*eff*_ for each radionuclide at different treatment stages was calculated using the formula^26^:

$$\:{R}_{eff}\:\left(\%\right)=\left[\frac{{C}_{Raw\:water}-{C}_{Stage}}{{C}_{Raw\:water}}\right]\times\:100$$

where C_*Raw water*_ represents the radionuclide concentration in the initial treatment stage and C_*Stage*_ is the concentration in the current stage. This calculation method enabled systematic evaluation of stage-specific performance and cumulative treatment effectiveness across the entire conventional treatment process.

**Table 1:** Summary of radionuclide concentrations with standard deviation and R_*eff*_ across treatment stages in Nile River water treatment plants.

Stage	Raw Water	Coagulation	*R*_eff_	Filtration	*R*_eff_	Final treated water	*R*_eff_
Rn-222 (Bq/L)							
Minimum	4.39 ± 0.22	3.11 ± 0.71	29.17%	1.61 ± 0.08	59.42%	1.09 ± 0.05	74.19%
Maximum	4.67 ± 0.23	3.29 ± 0.59		2.04 ± 0.10		1.25 ± 0.06	
Mean	4.51 ± 0.23	3.19 ± 0.06		1.83 ± 0.09		1.17 ± 0.06	
**Ra-226 (Bq/L)**							
Minimum	0.107 ± 0.01	0.091 ± 0.01	17.46%	0.082 ± 0.01	26.06%	0.080 ± 0.01	28.86%
Maximum	0.119 ± 0.01	0.097 ± 0.01		0.086 ± 0.01		0.083 ± 0.01	
Mean	0.115 ± 0.01	0.095 ± 0.01		0.085 ± 0.01		0.081 ± 0.01	
**Ra-228 (Bq/L)**							
Minimum	0.020 ± 0.002	0.014 ± 0.001	32.16%	0.012 ± 0.001	42.04%	0.011 ± 0.001	46.84%
Maximum	0.023 ± 0.002	0.015 ± 0.001		0.013 ± 0.001		0.012 ± 0.001	
Mean	0.021 ± 0.002	0.014 ± 0.001		0.012 ± 0.001		0.011 ± 0.001	
**K-40 (Bq/L)**							
Minimum	3.66 ± 0.35	3.17 ± 0.30	11.85%	3.19 ± 0.30	12.84%	2.86 ± 0.27	20.17%
Maximum	3.74 ± 0.36	3.33 ± 0.32		3.29 ± 0.31		3.10 ± 0.29	
Mean	3.71 ± 0.35	3.27 ± 0.31		3.23 ± 0.31		2.96 ± 0.28	

### Coagulation stage reduction effectiveness

The coagulation stage demonstrated differential effectiveness across radionuclide species, with R_*eff*_ ranging from 11.85% for K-40 to 32.16% for Ra-228. This variation directly correlates with the physicochemical properties of each radionuclide and the mechanisms of the coagulation process. The aluminum-based coagulants (typically alum) used in this stage create positively charged flocs that effectively capture negatively charged colloidal particles and associate radionuclides through charge neutralization and sweep flocculation mechanisms^40^. Ra-228 exhibited the highest R_*eff*_ (32.16%) during coagulation, despite being a soluble radionuclide. This behavior is attributed to its partial adsorption onto suspended particles—such as clay minerals and iron/manganese oxides—which are efficiently enmeshed within aluminum hydroxide flocs via sweep flocculation. In contrast, ²³²Th is virtually insoluble in natural waters (Ksp ≈ 10⁻⁴⁴ for Th(OH)₄) and remains associated with bed sediments; it is therefore not present in measurable concentrations in the aqueous phase and does not contribute to the activity detected in this study^41,42^. Ra-226, as an alkaline earth metal cation (Ra²⁺), exhibits moderate particle affinity, significantly lower than Th⁴⁺, and tends to remain dissolved in sulfate-poor waters like the Nile, where co-precipitation with barite or adsorption onto Fe/Mn oxides is limited^43^. K-40, being an alkali metal ion (K⁺), exhibited the lowest removal, primarily due to its predominant presence in the dissolved phase and weak adsorption onto flocs^40^. However, K-40 distribution can be influenced by sediment composition (e.g., illite or glauconite-rich clays), salinity, and anthropogenic inputs such as agricultural runoff—factors that may vary significantly across regions^17^. It should be noted that these removal mechanisms are strongly influenced by site-specific conditions, including source water chemistry, particle size distribution, and flocculation dynamics. While the behavior observed here reflects typical Upper Egyptian riverine systems, variations may occur in watersheds with different geological or anthropogenic characteristics.

### Filtration stage residual removal performance

The filtration stage provided additional radionuclide reduction, building upon the coagulation process to achieve cumulative R_*eff*_. This stage employs specialized sand filters with fine mesh underdrains that physically retain flocs and residual particulate matter not captured in previous stages. By the end of the filtration stage, the cumulative removal efficiency for Rn-222 reached 59.42%, 26.06% for Ra-226, 42.04% for Ra-228, and 12.84% for K-40. The significant increase in Rn-222 removal during filtration (from 29.17% to 59.42%) cannot be attributed solely to particulate capture, as Rn-222 is a volatile gas. This enhanced removal likely results from incidental degassing during filtration, where hydraulic shear, media contact, and transient air pockets within the filter bed promote Rn-222 volatilization—even in nominally closed filter systems. The multi-layered sand filter system provides extended contact time and increased surface area for gas transfer, making filtration a significant contributor to Rn-222 removal in conventional treatment trains. Ra-228 maintained its position as the most effectively removed radionuclide (42.04% cumulative efficiency), demonstrating the filtration system’s capability to capture fine particulate matter and associated radionuclides. The relatively high efficiency suggests that the coagulation-flocculation process successfully formed stable flocs containing Ra-228-associated particles that were efficiently retained by the filter media. The minimal additional removal of K-40 during filtration (increasing from 11.85% to 12.84%) confirms its predominantly dissolved state and limited association with removable particles. While K-40 generally exhibits low R_*eff*_ due to its high solubility and ionic nature in aqueous systems, this behavior can vary depending on the geochemical matrix of the source water. In environments where suspended sediments are rich in K-40-bearing minerals—such as illite or glauconite—the potential for particulate-associated transport increases, which may enhance removal during coagulation-filtration stages^17^. However, in the Upper Nile River system, the predominance of dissolved-phase K-40 suggests limited association with removable particles under current treatment conditions.

### Final treated water quality achievement

**Fig. 3:**
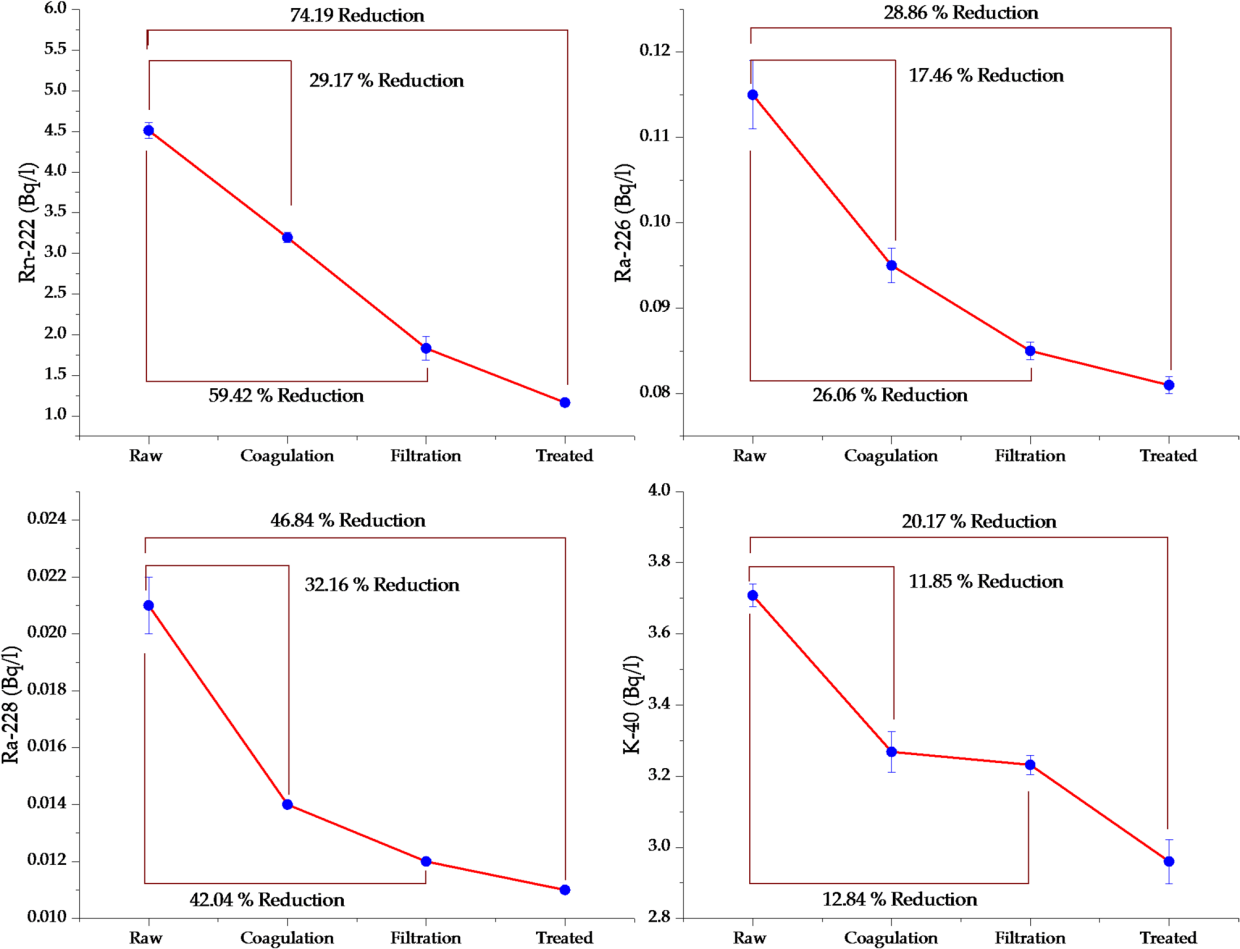
Stage-by-stage radionuclides concentration reduction and R_*eff*_ patterns.

By the final treated water stage, the cumulative R_*eff*_ reached 74.19% for Rn-222, 28.86% for Ra-226, 46.84% for Ra-228, and 20.17% for K-40. These cumulative efficiencies demonstrate the effectiveness of the multi-barrier treatment approach employed by conventional treatment plants in Upper Egypt. The highest overall R_*eff*_ for Rn-222 (74.19%) reflects the cumulative effect of incidental degassing during coagulation (due to chemical mixing), filtration (through media contact and hydraulic turbulence), and final storage in contact tanks, where residual volatilization occurs before distribution. While the cumulative R_*eff*_ reflects the treatment process performance, actual human exposure may be further reduced by post-distribution factors such as short residence time in the distribution network and household practices like boiling or aeration, which promote additional Rn-222 volatilization. Ra-228’s second-highest removal (46.84%) underscores the effectiveness of particulate capture mechanisms throughout the treatment process. The consistent performance across all stages indicates that Ra-228 is partially associated with suspended particles, enabling its co-removal with flocs rather than dissolved species. The Ra-226 removal of 28.86% suggests that a sizable portion exists in dissolved form or is associated with particles that are not completely captured by conventional treatment. This moderate efficiency is typical for divalent cations in surface water treatment systems^17,44^. K-40’s lowest R_*eff*_ (20.17%) is expected given its ionic nature and high solubility. The minimal removal indicates that K-40 behaves primarily as a dissolved species that is not significantly affected by conventional water treatment processes. The stage-by-stage analysis reveals that each treatment barrier contributes meaningfully to overall radionuclide reduction, with coagulation and filtration providing the primary removal mechanisms for radionuclides that exhibit partial affinity for suspended particles, such as Ra-228, while volatilization processes dominate Rn-222 removal. Figure 3 illustrates the distinct removal patterns of each radionuclide through the multi-barrier treatment process. The visualization clearly shows that Rn-222 experiences the most substantial reduction, while Ra-228 maintains consistent particulate capture efficiency. Ra-226 shows moderate removal, with only a marginal 2.8% additional reduction between filtration and final treated water, indicating effective process stabilization by the end of filtration. Multiple factors contribute to the observed variations in radionuclide removal. Water chemistry parameters—including pH, ionic strength, and dissolved organic carbon—affect radionuclide speciation and treatment effectiveness. Alum coagulation is most effective within a specific pH range (typically 5.5–8.5), while dissolved organic matter can be complex with radionuclides. Particle size distribution plays a crucial role; radionuclides associated with fine colloidal particles (< 1 μm) are more challenging to remove than those bound to larger suspended particles. Hydrodynamic conditions such as turbulence and residence time significantly influence Rn-222 removal through degassing mechanisms. It should be noted that radionuclide behavior during conventional treatment is highly dependent on site-specific factors. While our findings reflect typical performance in river-fed surface water plants, they may not directly apply to groundwater systems or watersheds influenced by agricultural runoff. This site-specific nature underscores the need for localized radiological assessments, particularly in areas with granitic bedrock or known uranium anomalies, where raw water activity may exceed the low background levels observed in the Upper Nile^9,17^.

## Radiological health risk assessment

The annual effective doses (D_*an*_) due to the ingestion of Nile-derived drinking water were calculated for 3 age groups: adults, children, and infants. The assessment considered only Rn-222, Ra-226, and Ra-228, as K-40 was excluded based on its biological regulation in the human body. K-40 is an essential element whose absorption is tightly controlled by homeostatic mechanisms, primarily through dietary intake rather than drinking water^31^. Additionally, its dose conversion factor (D_*cf.*_) is exceptionally low (6.2 × 10⁻⁹ Sv/Bq for adults), resulting in a negligible radiological contribution compared to other radionuclides. It should be noted that the observed R_*eff*_, such as 28.86% for Ra-226, reflect the low initial activity of Nile River water (0.115 Bq/L), which is substantially lower than levels reported in high-background areas like Nasser Lake^9^ or granite-rich regions of Finland and Portugal (> 1 Bq/L)^17,45^. In such high-activity settings, advanced treatment methods (e.g., ion exchange, reverse osmosis, aeration) are often employed to meet safety standards. In contrast, conventional coagulation-filtration remains adequate for the Upper Nile context, where raw water radioactivity is already within safe limits. The D_*cf.*_ values used in this study are based on the recommendations of the International Commission on Radiological Protection (ICRP)^46^, which account for both radionuclide-specific decay properties and age-related physiological differences. Age-specific water ingestion rates were based on local consumption surveys in Egypt and conservative exposure assumptions: 500 L/year for adults, 350 L/year for children (ages 1–12), and 150 L/year for infants (< 1 year), reflecting typical domestic water use patterns in the study region^9^. For dosimetric purposes, the dose conversion factors for ²²⁶Ra and ²²⁸Ra are identical across all age groups, as recommended by the ICRP^46^, because both radionuclides behave identically in the human body: as alkaline earth metals (Ra²⁺ ions), they are chemically similar to calcium, leading to comparable absorption, distribution, and long-term retention primarily in bone tissue, and thus the radiological risk is governed by their shared biokinetic behavior rather than differences in their decay chains; the dose conversion factors used in this study are as follows: for Rn-222, 1.0 × 10⁻⁸ Sv/Bq for adults, 2.0 × 10⁻⁸ Sv/Bq for children (1–12 years), and 7.0 × 10⁻⁸ Sv/Bq for infants (< 1 year); for both Ra-226 and Ra-228, 2.8 × 10⁻⁷ Sv/Bq for adults, 6.2 × 10⁻⁷ Sv/Bq for children, and 2.3 × 10⁻⁷ Sv/Bq for infants. These values are consistent with WHO^40^ and the United Nations Scientific Committee on the Effects of Atomic Radiation (UNSCEAR)^47^ guidance for ingestion dose assessment from drinking water. These values were applied using the standard international equation for calculating D_*an*_^9,12^:

$$\:{D}_{an}\:({\upmu\:}\mathrm{S}\mathrm{v}/\mathrm{y})=\sum\:({C}_{i}\times\:{D}_{cfi}\times\:W)\times\:{10}^{6}$$

where *C*_*i*_ is the concentration of radionuclide *i* in treated water (Bq/L), D_*cf. i*_ is the age-specific dose conversion factor (Sv/Bq), and W is the annual water consumption rate (L/year). Using this formula, the D_*ann*_ for all ten treatment plants were calculated. Table 2 summarizes the minimum, maximum, and average D_*ann*_ across all plants for each age group. The complete table is provided with supplementary materials (Table S.3). These tables allow for detailed comparisons between facilities and support comprehensive evaluation of treatment performance across different operational conditions.

**Table 2:** Annual effective doses due to ingestion of Nile-derived drinking water and comparison with international limits.

Age Group	Annual Effective Dose (µSv/y)			
	Minimum	Maximum	Average	Screening Level [40] [46]
Raw Water intake				
Adults	40.2	43.3	41.6	100
Children	59.0	63.6	61.0	
Infants	50.9	53.9	52.0	
Treated Water intake				
Adults	18.5	19.1	18.8	100
Children	27.8	28.7	28.3	
Infants	14.7	16.3	15.4	

All screening levels are based on the international guidance of a maximum annual effective dose of 100 µSv from a single source, as adopted by WHO^40^ and the ICRP^46^. Although age-specific dose conversion factors were used in calculations, the same screening level applies across all age groups.

### Comparison with international safety standards

The calculated D_*ann*_ for the 3 age groups demonstrate significant radiological risk reduction through conventional water treatment processes. As shown in Table 2, raw water produces average D_*ann*_ of 41.6 µSv/year for adults, 61.0 µSv/year for children, and 52.0 µSv/year for infants. Following treatment, these doses decrease to 18.8 µSv/year, 28.3 µSv/year, and 15.4 µSv/year, respectively, representing dose reductions of 54.8%, 53.6%, and 70.4%, respectively. These treated water doses remain well below the internationally adopted screening level of 100 µSv per year for a single source of radiation in drinking water, as recommended by WHO^40^ and the IAEA^48^. This compliance confirms that Egyptian treatment facilities effectively maintain radiological safety standards across all consumer age groups.

### Health risk evaluation for treated water consumption

**Fig. 4:**
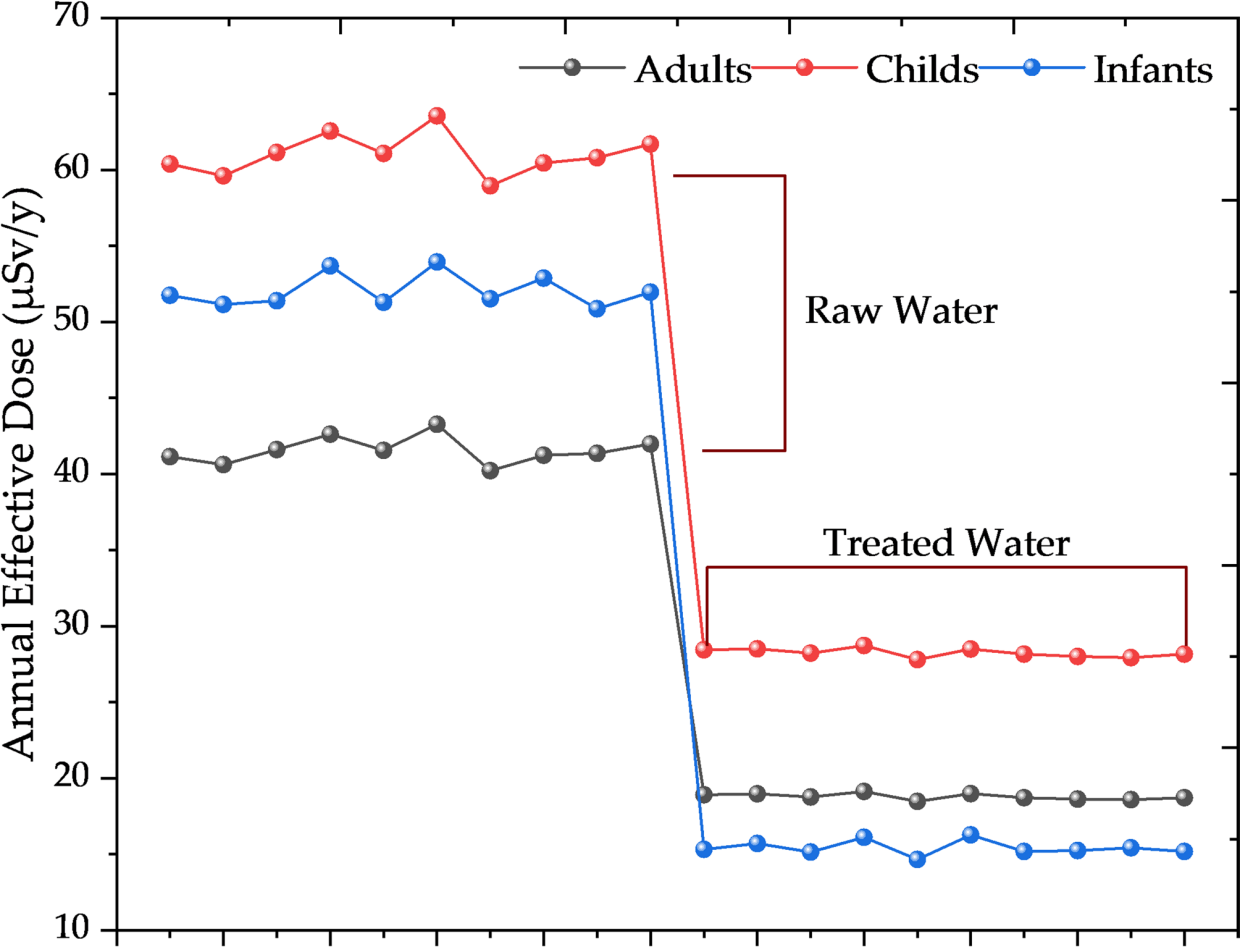
Radiological dose reduction effectiveness across age groups in Nile River drinking water treatment.

The health risk assessment reveals that conventional treatment processes provide adequate protection for Nile-dependent populations. The calculated annual doses correspond to negligible excess lifetime cancer risks (well below 0.01%), representing a minimal increment over the baseline lifetime cancer risk of approximately 40%, confirming that long-term consumption of treated Nile River water does not pose a significant radiological hazard. The differential dose reductions among age groups reflect both varying water consumption patterns and age-specific D_*cf.*_. Infants exhibit the highest dose reduction (70.4%) because Rn-222 contributes disproportionately to their total dose—due to their elevated D_*cf.*_ for Rn-222 (7.0 × 10⁻⁸ Sv/Bq)and Rn-222 is the most effectively removed radionuclide (R_*eff*_ = 74.19%). In contrast, adults and children receive a larger relative contribution from Ra-226 and Ra-228, which are less efficiently removed, resulting in lower overall dose reductions (Fig. 4).

### Long-term exposure implications

Long-term consumption of treated Nile River water poses minimal radiological health risks, with doses from the final treated water remaining consistently below 28.3 µSv/year, or 28.3% of the international screening level of 100 µSv/year across all ten plants in Upper Egypt^47^. It should be noted that while conventional treatment effectively reduces radionuclide levels in finished water, the process may concentrate natural radioactivity in treatment residuals such as sludge and spent filter sand. In regions with elevated raw water activity (e.g., uranium-rich aquifers), these by-products can accumulate radionuclides to levels warranting regulatory oversight as Technologically Enhanced Naturally Occurring Radioactive Material (TENORM)^17,49–51^. In the Upper Nile context, where raw water activity is relatively low (Ra-226: 0.115 Bq/L; Ra-228: 0.021 Bq/L), the radiological burden on residuals is expected to remain minimal. Nevertheless, future monitoring of sludge and filter media is recommended to ensure safe disposal practices and prevent potential secondary contamination.

## Conclusion

This study demonstrates that conventional multi-barrier treatment in Upper Egypt effectively mitigates natural radionuclides in Nile River water, ensuring public health protection. The process achieved differential removal efficiencies, highest for Ra-228 (46.84%), which exhibits partial affinity for suspended particles, and the volatile Rn-222 (74.19%), and lowest for the dissolved ionic K-40 (20.17%), highlighting the direct influence of radionuclide speciation on treatment effectiveness (R_*eff*_). The sequential treatment stages functioned synergistically: coagulation removed radionuclides that associate with suspended particles, particularly Ra-228, filtration enhanced particulate capture and Rn-222 degassing, and the final holding stage allowed for residual Rn-222 degassing. This multi-barrier approach reduced annual effective doses by 53.6–70.4% across age groups, resulting in final doses (15.4–28.3 µSv/year), which represent 15.4–28.3% of the 100 µSv/year international screening level, well within acceptable radiological safety margins. While validating the adequacy of existing infrastructure for current Nile water quality, the results underscore inherent limitations in removing dissolved radionuclides. These insights provide a basis for future process optimization should source water quality change. The study offers evidence-based guidance for monitoring and safety standards in Nile-dependent communities and similar regions globally. Future studies should quantify radionuclide accumulation in treatment residuals (e.g., sludge and spent filter media) to inform safe disposal protocols and prevent secondary environmental exposure.

## Supplementary Information

Below is the link to the electronic supplementary material.

Supplementary Material 1

## Data Availability

The datasets generated and analyzed during the current study are available from the corresponding author on reasonable request.
